# Association between user engagement and clinical outcomes in smartphone apps for depression and anxiety: A systematic review and meta-analysis

**DOI:** 10.1016/j.psychres.2025.116864

**Published:** 2025-11-25

**Authors:** Jake Linardon, John Torous, Mariel Messer, Claudia Liu, Imogen H. Bell, Jennifer Nicholas, Simon B. Goldberg

**Affiliations:** a SEED Lifespan Strategic Research Centre, School of Psychology, Faculty of Health, Deakin University, Geelong, VIC, Australia; b Division of Digital Psychiatry, Beth Israel Deaconess Medical Center, Harvard Medical School, Boston, MA, United States; c Orygen, Parkville, VIC, Australia; d Centre for Youth Mental Health, University of Melbourne, Melbourne, VIC, Australia; e Department of Counseling Psychology and Center for Healthy Minds, University of Wisconsin, Madison, WI, United States

**Keywords:** Smartphones, Depression, Anxiety, Meta-analysis, Engagement

## Abstract

Apps targeting symptoms of depression and anxiety have a growing evidence base for their efficacy, yet it remains unclear whether increased user engagement is necessary to enhance their benefits. This systematic review and meta-analysis examined the current evidence on the association between engagement and clinical outcomes in trials of depression and anxiety apps. We included original or secondary analyses of randomized trials of apps delivered to participants with elevated depression or anxiety that reported engagement-outcome relationships. Studies were identified through a previous systematic review and an updated search. Twenty-eight studies met inclusion criteria for the systematic review, and 13 were included in the meta-analysis. Qualitative synthesis revealed heterogeneity in how engagement-outcome associations were reported: >40 different engagement metrics were identified, and 57 % of studies examined multiple metrics as predictors. Averaging across engagement and outcome variables, a significant pooled effect was found (*r* = 0.16; 95 % CI= 0.09, 0.21), indicating that greater engagement was linked to larger symptom improvement. This effect remained significant following publication bias adjustment and when assuming a zero effect for seven studies that reported non-significant associations but no accompanying data (*r* = 0.11, 95 % CI= 0.06, 0.16). Significant effects were also observed when modeling specific engagement metrics, symptom outcomes, and app characteristics, although few studies contributed to these analyses. Engagement may play a small role in symptom improvement within depression and anxiety apps. Findings highlight the need for better reporting standards, including which theory-driven engagement metrics should be routinely reported in future research.

## 1. Introduction

Depression and anxiety are leading causes of disability worldwide (Santomauro et al., 2021), yet most individuals affected go untreated (World Health Organization, 2022). Interventions translated for delivery via smartphone applications (apps) can increase access to evidence-based psychological treatments due to their low cost, widespread availability, and ability to be used discreetly (Torous et al., 2021, 2025). This discreet access can help reduce stigma-related barriers, which remain a major deterrent to seeking face-to-face care (Kazdin, 2017). Apps can also incorporate varying degrees of personalisation, ranging from simple surface-level tailoring (e.g., use of the individual’s name) to more substantive adaptations, such as recommending specific modules or exercises based on users’ reported symptoms, preferences, or progress (Linardon and Torous, 2025). Their portability allows individuals to access therapeutic content and apply learned skills in real-world contexts and moments of need, supporting the integration of treatment strategies into daily life.

While evidence supports the efficacy of apps for depression and anxiety as both a stand-alone option or an adjunct to traditional care (Linardon, Torous, et al., 2024), sustained user engagement remains a key concern (Linardon and Fuller-Tyszkiewicz, 2020; Torous et al., 2018). Clinical trials show that up to 60 % of participants fail to complete all prescribed modules, and as many as 70 % disengage within a few weeks of use (Peake et al., 2024; Sirbu et al., 2025). Engagement issues are even more pronounced in naturalistic settings, with seminal work indicating that 30-day retention rates for popular mental health apps can be as low as 3 %, and daily active user rates hover around just 4 % (Baumel et al., 2019).

These findings have prompted a new generation of research aimed at investigating theories of engagement (Nahum-Shani et al., 2022) and developing effective engagement strategies, with promising evidence found for personalised push notifications (Bidargaddi et al., 2018), gamification techniques (Looyestyn et al., 2017), digital navigators (Perret et al., 2023), and peer support components (Fortuna et al., 2019). However, it may be premature to invest heavily in testing or optimising engagement strategies without first establishing whether increased engagement is indeed necessary to maximise the clinical benefits of mental health apps. Previous reviews of engagement in mental health apps have primarily focused on its reporting, measurement, and validity (Ng et al., 2019; Torous et al., 2020, 2018), but have not yet systematically examined the extent to which engagement – or specific engagement metrics – are associated with clinical outcomes. On the other hand, prior reviews of web-based mental health interventions highlight the complexity of the assumed engagement-outcome relationship, suggesting that the strength of the association may depend on how engagement is measured (Donkin et al., 2011; Gan et al., 2021). For example, greater program completion and total website exposure appear to be consistently linked to improved outcomes for depression, whereas metrics such as number of logins, self-reported task completions, time spent online, and pages viewed show no consistent association (Donkin et al., 2011).

While earlier reviews addressing this question offer important insights into the potential impact of user engagement on mental health outcomes, their exclusive focus on web-based interventions limits the generalisability of findings to smartphone apps targeting depression and anxiety. Web-based programs are typically accessed via desktop computers and follow a structured, modular-based format that emphasises psychoeducation and sequential learning (Andersson, 2024). However, over the past five years, this distinction has become less pronounced, as many digital mental health programs are now designed with a mobile-first approach and can be accessed on smartphones through responsive websites or progressive web apps, which often replicate much of the app experience. The primary differences tend to relate to native functionality, such as push notifications, offline access, and integration with smartphone sensors, which can influence both engagement opportunities and how engagement is measured (Torous et al., 2019). For example, whereas web program trials have historically reported metrics such as module completion or time on site (Donkin et al., 2011), app trials often include additional metrics like number of opens, session duration, and activity frequency (Ng et al., 2019). Given these overlapping but not identical design and functionality characteristics, findings from earlier web-based programs cannot be assumed to fully generalise to modern app-based interventions, highlighting the need for research that specifically examines engagement-outcome associations in trials of mental health apps.

The purpose of this review is to systematically examine the association between engagement and clinical outcomes in randomized controlled trials (RCTs) of mental health apps targeting depression and anxiety. More specifically, we aim to (1) synthesise research reporting on the associations between engagement and outcomes; (2) characterize the types of engagement metrics assessed in relation to predicting outcomes; and (3) quantify the extent of the engagement-outcome association through meta-analytic techniques, and explore whether different metrics of engagement (e.g., days of use, number of activities completed, time spent on app, etc.) are more strongly related to symptom change.

## 2. Method

### 2.1. Identification and selection of studies

We pre-registered this review in the Open Science Framework repository (https://osf.io/xbh96) and adhered to the PRISMA guidelines (Page et al., 2021). Pre-registration was submitted after the search was conducted but prior to the full-text screening and data extraction process. We first identified potentially eligible studies from a recent 2024 review on adverse events in clinical trials of mental health apps (Linardon, Fuller-Tyszkiewicz, et al., 2024). This review captured all available randomized trials on depression and anxiety apps up to 2024 that would meet eligibility for this research (search terms in the supplementary materials). We then updated the search by searching the Medline and PsycINFO databases from January 2024 to March 2025 using the following combinations of key terms: smartphone*” “mobile phone” “mobile app*” “iphone” “android” “mhealth” “m-health” “mobile device*” “mobile-based” “mobile health” “tablet-based” app-based app-supported app-assisted AND random* “clinical trial” AND anxiety, anxious, phobia,* panic agoraphobia “mental health” “mental illness*” “depress*” “affective disorder*” “mood disorder*” mood. A secondary search strategy was employed by screening the reference lists of included trials and relevant reviews in this area. Furthermore, because analyses on engagement-outcome associations are often conducted *post hoc* and may be reported in secondary publications rather than the original trial reports, we also reviewed all available records that cited each trial of depression/anxiety apps to ensure that no relevant companion studies were missed

Eligibility criteria were defined using the PICO framework:

**Population:** Individuals with elevated depression and/or anxiety, established through a diagnostic interview, scoring above a cut-off on a validated self-report scale, or by participant self-report. In this review, “elevated” refers to baseline symptom levels that either exceeded established clinical or subclinical cut-off scores on validated measures or were self-reported by participants as clinically significant difficulties, indicating elevated symptomatology rather than change from a prior timepoint.
**Intervention:** Native mental health smartphone applications designed to address symptoms of depression and/or anxiety. Apps could be delivered as either stand-alone interventions or as adjuncts to traditional clinical services.
**Comparator:** Any comparator condition, including waitlist, usual care, attention control, or alternative app-based interventions.
**Outcome:** Clinical outcomes related to depression and/or anxiety and their association with objective app engagement metrics (e.g., number of logins, days of use, activities completed). Studies relying solely on retrospective self-reports of engagement were excluded. Two researchers performed the screening at the full-text stage to determine whether the paper met full inclusion criteria. Inter-rater agreement was excellent (κ = 0.97).

### 2.2. Risk of bias and data extraction

Risk of bias was assessed using five domains from the Cochrane Risk of Bias tool (Higgins et al., 2019): random sequence generation; allocation concealment; blinding of participants or personnel, blinding of outcomes; and completeness of outcome data. Each domain received either a high, low or unclear rating. We also extracted the following information from eligible studies: target sample; sample selection criteria; sample size; app name; app orientation (e.g., CBT vs non-CBT – an app was coded as CBT if it explicitly reported being based on CBT principles or if its central therapeutic component involved cognitive restructuring); presence of key in-app features (guidance, symptom monitoring technology, and chat-bot); treatment delivery mode (stand-alone or adjunctive); type of control group; engagement metrics and their operationalization; outcome variables; follow-up length; engagement-outcome relationship data; and a summary of findings. Two researchers performed data extraction, with minor discrepancies (κs > 0.82) resolved through consensus.

### 2.3. Analytical approach

We first performed a narrative synthesis of findings regarding associations between engagement metrics and clinical outcomes. Findings were synthesized overall and then by target symptom outcome. We focused on the proportion of significant and non-significant relationships identified in this qualitative synthesis.

Meta-analyses were then performed to quantify associations between engagement and symptom improvement within the app arm among those trials that reported sufficient data to calculate effect sizes (*k* = 13). Pearson’s correlation coefficient (*r*) was selected as the measure of effect size, with values of 0.1 considered small, 0.3 considered medium, and 0.5 considered large (Cohen, 1992). One trial provided beta weights (Mohr et al., 2019), which were then converted to *r* based on prior recommendations (Peterson and Brown, 2005). For the two trials that dichotomized engagement (high vs. low; Greer et al., 2019; Mantani et al., 2017), standardized mean differences were calculated first and then converted to *r* through standard methods implemented in the Comprehensive Meta-Analysis (CMA) software. Correlation coefficients were transformed prior to analyses using Fisher’s Zr-transformation so that each effect size could be weighted by its inverse variance (Lipsey and Wilson, 2001). These effect sizes were converted back into standard correlation coefficients when reporting results. All correlations were standardized such that a positive coefficient indicated that greater engagement was associated with larger symptom improvement.

A total pooled effect for the association between engagement and symptom change was computed by first aggregating within-study correlations across engagement metrics and outcome measures at post-test. This within-study averaging was conducted using CMA, which applies a sample size-weighted approach before pooling effects in the overall meta-analysis. Engagement metrics included in the meta-analyses encompassed number of days of use, number of activities/tasks completed, number of app sessions, and time spent on the app, as these were consistently reported among the 13 trials eligible for analysis. We then conducted a series of sensitivity analyses, calculating pooled effects separately (where feasible) for those specific engagement metrics and for specific symptom outcomes.

Random effects models were used. Heterogeneity was assessed through the I^2^ statistic, which quantifies heterogeneity revealed by the Q statistic and reports how much overall variance (0–100 %) is attributed to between-study variance (Higgins and Thompson, 2002). We employed the trim-and-fill procedure (Duval and Tweedie, 2000) to evaluate the impact of potential publication bias. We considered additional publication bias tests (e.g., precision-effect test and precision-effect estimate with standard errors [PET-PEESE]), but did not pursue this due to poor performance of these methods in the context of small numbers of studies (e.g., *k* < 20), especially when the true effect is small (Stanley, 2017). However, in a conservative sensitivity analysis, we assumed an effect size of *r* = 0.00 for studies that reported finding a nonsignificant association between engagement and outcomes but did not report an exact effect size or other usable data (e.g., the paper merely reported that associations were n.s or that *p* > .05). Table 1 outlines these studies.

Pre-registered subgroup analyses were not conducted given the limited number of studies available for meta-analysis, but sensitivity analyses were performed by computing effects for specific trial features to see if the total effects were robust under various conditions.

## 3. Results

**Fig. 1.**
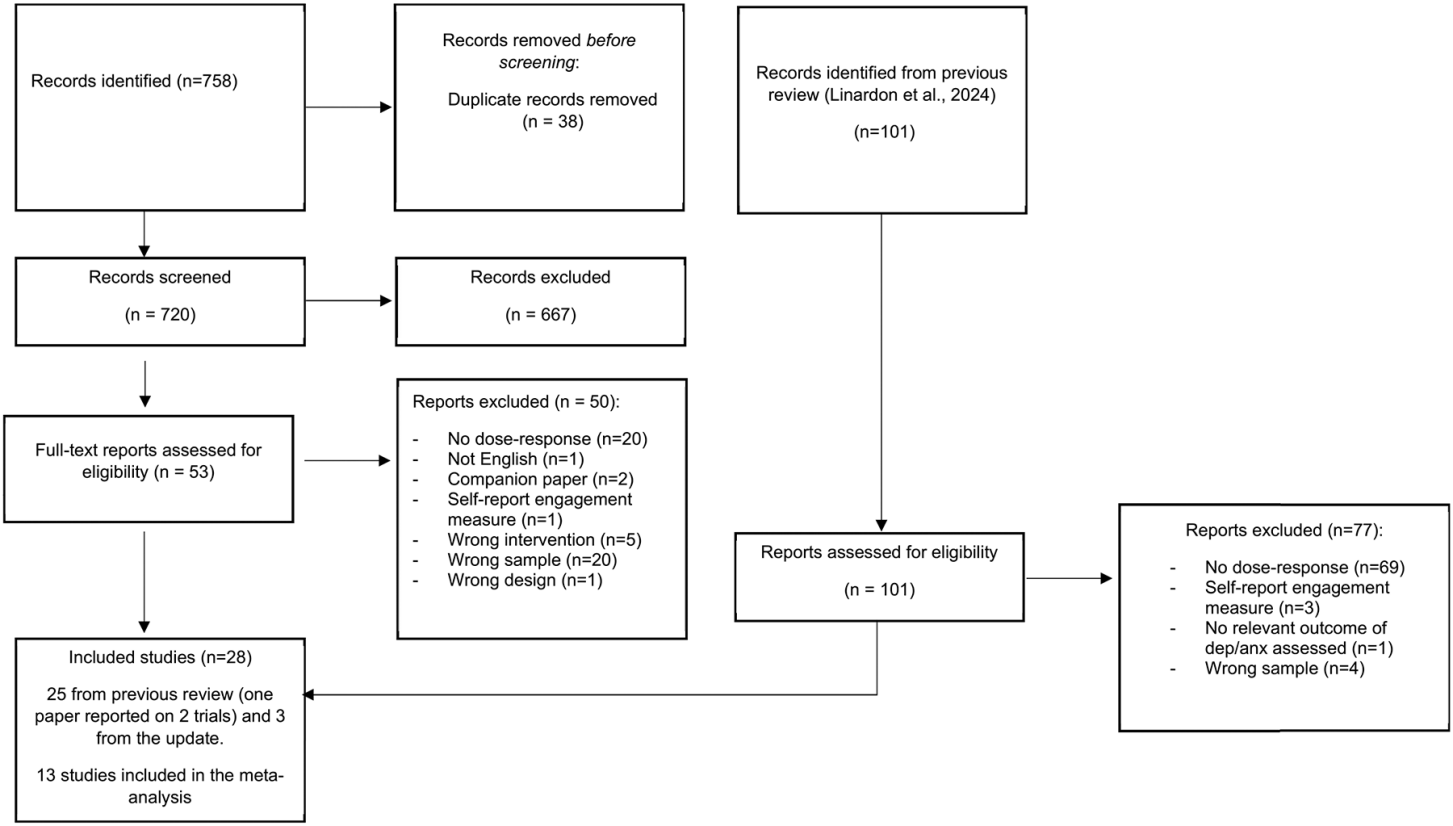
Flowchart of literature search.

A flowchart of the literature search is presented in Fig. 1. Twentyfour studies from the prior review met the full inclusion criteria, and an additional four studies were identified through the updated search, bringing the total to 28 studies included in the systematic review. Four of these trials conducted secondary analyses on dose-response associations reported in companion publications. Thirteen studies provided sufficient to calculate effect sizes for meta-analysis (Table 1). Seven studies reported a non-significant engagement-outcome association but did not provide data necessary to calculate effects (Araya et al., 2021; Catuara-Solarz et al., 2022; Miklowitz et al., 2023; Newman et al., 2021; Oh et al., 2020; Stiles-Shields et al., 2018; Stiles-Shields et al., 2024; Tighe et al., 2017), so these were included in the sensitivity meta-analyses that assumed an *r* = 0.00.

### 3.1. Study characteristics

Table 1 presents the characteristics of the included studies. Samples comprised participants with elevated depression (*k* = 13), generalized anxiety (*k* = 4), mixed anxiety and depression^1^ (*k* = 5), social anxiety (*k* = 3), panic (*k* = 1), and specific phobic (*k* = 2) symptoms. Most trials screened participants based on self-report (*k* = 18) rather than diagnostic interviews (*k* = 10). There were 34 app conditions in total, most of which were based on CBT (*k* = 27), had symptom tracking features (*k* = 21), and were delivered in guided self-help format (*k* = 19). Few trials incorporated chatbot technology (*k* = 2). More trials delivered the app as a stand-alone intervention (*k* = 22) rather than an adjunct to more intensive treatment (*k* = 6). Waitlists were the most common type of control conditions (*k* = 12). The total sample size randomized ranged from 30 to 1312. Risk of bias ratings varied across studies. Twenty-one trials met criteria for adequate sequence generation, 11 met criteria for adequate allocation concealment, eight met criteria for proper participant blinding (e.g., in a way ensuring that participants were not aware of the condition they were allocated to), 27 used blinding outcome assessors or self-report outcome measures, and 18 met criteria for appropriate handling of missing data. Two trials were identified as low risk of bias across all criteria, eight met four, 11 met three, three met two, and four trials met one of the criteria.

### 3.2. Narrative synthesis

#### 3.2.1. All studies

There was variability in the number of engagement metrics tested for its association with outcomes. The minimum and maximum number of metrics reported was one and 10, with most trials (*k* = 13) only reporting one metric. The mean number of metrics reported from 28 studies was 2.3 (SD = 2.0).

Table 2 presents the number of statistically significant and non-significant associations between different engagement metrics and symptom outcomes across included studies. Engagement metrics were divided into five broad categories: activity/task completion; days of use; time of use; number of sessions/opens; and “other”. Across all samples, most studies used at least one measure of activity/task completion (*k* = 18), with 34 associations with symptom improvement tested. Of these, eight were statistically significant and in the expected direction. Number of app opens/sessions was used in nine studies, with 13 associations tested, three of which were significant and in the expected direction. Days of app use and time of use were, respectively, used in six and seven studies, with seven associations tested each. Of these, only one association was statistically significant for both metrics. “Other” engagement metrics included, for example, time to last use, module completions, and click rate, of which there were three of 12 associations that were statistically significant. Below, we provide a more detailed narrative synthesis by target symptom.

#### 3.2.2. Depression

Nineteen studies used a measure of depressive symptoms as an outcome; of these, thirteen sampled participants with elevated depression (Araya et al., 2021; Bastiaansen et al., 2022; Guo et al., 2020; Kulikov et al., 2023; Mantani et al., 2017; Miklowitz et al., 2023; Raevuori et al., 2021; Six et al., 2022; Stiles-Shields et al., 2018; Tighe et al., 2017; Yatziv et al., 2024; Zuccolo et al., 2024), five sampled a transdiagnostic population with mixed depression and/or anxiety (Bell et al., 2023; Graham et al., 2020; Moberg et al., 2019; Mohr et al., 2019; Colleen Stiles-Shields et al., 2024), and one sampled participants with elevated anxiety but included depression as a secondary outcome (Greer et al., 2019). Table 2 shows that there were 42 associations tested between engagement and changes in depression, most of which comprised the operationalization of in-app activity/task completions (*n* = 23). Across all engagement metrics studied, nine associations were statistically significant and in the expected direction, including five related to in-app task completions. Two of five significant associations were in relation to number of app sessions. Thirty-three associations were statistically non-significant. No studies reported a significant association in a negative direction.

#### 3.2.3. Generalized anxiety

Ten studies used a measure of generalized anxiety symptoms as an outcome; four sampled participants with elevated general anxiety (Catuara-Solarz et al., 2022; Greer et al., 2019; Newman et al., 2021; Roy et al., 2021), five sampled a mixed depression and/or anxiety sample (Bell et al., 2023; Graham et al., 2020; Moberg et al., 2019; Mohr et al., 2019; Stiles-Shields et al., 2018), and one reported generalized anxiety as a secondary outcome in a sample with depression (Raevuori et al., 2021). There were 29 associations tested between engagement and changes in generalized anxiety. Most of these operationalized engagement in terms of number of in-app activities (*n* = 10) or sessions (*n* = 8). Across all metrics, only two significant (and in the expected direction) associations were observed – one when engagement was operationalized as the number of downloads and the other when it was operationalized as the number of modules completed.

#### 3.2.4. Social anxiety

Three studies used a measure of social anxiety symptoms as an outcome (Saulnier et al., 2023; Schwob and Newman, 2023; Stolz et al., 2018), all in samples screened for elevated social anxiety. Thirteen engagement-outcome associations were tested, eight of which operationalized engagement as number of in-app activities. Four associations were statistically significant and in the expected direction: three were in relation to number of in-app activities and the fourth was in relation to number of app lessons completed. The other nine associations were non-significant.

#### 3.2.5. Specific phobia

Two trials sampled participants with a specific phobia (Donker et al., 2019; Lacey et al., 2023). One trial operationalized engagement in terms of time of app use and number of activities completed (Donker et al., 2019), while the other operationalized engagement as hours of app use (Lacey et al., 2023). The only significant association identified was for time of app use, with longer use predicting greater reduction in phobic symptoms.

#### 3.2.6. Panic

One trial sampled participants with panic disorder (Oh et al., 2020). No significant associations were found between time spent on the app and total days of use with changes in panic symptoms.

### 3.3. Meta-analysis

Table 2 presents the results from the meta-analyses. A small but significant total pooled effect size was observed from 13 studies (*r* = 0.16; 95 % CI = 0.09, 0.21), indicating that greater engagement was associated with larger symptom change. Heterogeneity was absent (I^2^ = 0 %). There were no outliers (Table 3).

The impact of publication bias was examined using the trim-and-fill procedure. The pooled effect size remained significant and similar in magnitude when applying the trim-and-fill procedure (*r* =. 14; 95 % CI = 0.07, 0.21). However, in a conservative sensitivity analysis that assumed non-significant effects were *r* = 0.00 for seven studies that reported a non-significant relationship without accompanying effect size data, the pooled effect was smaller but still significant (*r* = 0.11, 95 % CI = 0.06, 0.16).

Sensitivity analyses show that the effect remained robust when restricting the analyses to specific engagement and symptom outcome variables and when removing one trial with a high risk of bias rating. Significant effects were observed when engagement was operationalized as the number of activities completed (*k* = 9; *r* = 0.19; 95 % CI = 0.11, 0.27), number of sessions completed (*k* = 6; *r* = 0.12; 95 % CI = 0.05, 0.20), and time on the app (*k* = 3; *r* = 0.17; 95 % CI = 0.01, 0.31), but not from two studies that assessed engagement as days of use (*r* = 0.11; 95 % CI = 0.15, 0.37). Significant effects were also observed when changes in depression (*k* = 9; *r* = 0.14; 95 % CI = 0.07, 0.21), generalized anxiety (*k* = 6; *r* = 0.09; 95 % CI = 0.01, 0.18), and social anxiety (*k* = 4; *r* = 0.25; 95 % CI = 0.08, 0.40) were used as outcomes.

Effects also remained stable when limiting the analyses to specific app features. Significant, positive associations between engagement and symptom change were found for guided and unguided apps, apps with symptom monitoring technology, and apps delivered as either a standalone intervention option or an augmentation to traditional care (*r*s = 0.15 to 0.19).

## 4. Discussion

This review of 28 studies synthesized empirical research investigating associations between engagement and clinical outcomes in randomized trials of mental health apps targeting depression or anxiety. Findings from our narrative synthesis highlight the complexities of the engagement-outcome association. Certain engagement metrics, particularly the number of activities or tasks completed, were more consistently linked with symptom improvement, especially for depression and social anxiety. Nonetheless, the heterogeneity in engagement operationalization, number of metrics assessed, and variability in sample size and study quality limited the ability to draw definitive conclusions from our qualitative synthesis regarding the role of engagement in symptom improvement.

Finding in-app activity completions to be one of the more consistent outcome predictors contrasts with Donkin et al.’s (2011) review of web-based interventions, which found no reliable association between these metrics and mental health symptom improvement. Importantly, their review relied on self-reported (not objective) activity completion, limiting the reliability of its conclusions. This is because while web-based platforms often capture objective metrics such as logins and module completion, they are limited in tracking skill use in daily life (Andersson, 2016). Unlike smartphone apps, they lack the portability and sensor integration needed to monitor behaviour in real-world settings. In contrast, apps routinely capture detailed user data in situ, allowing for more accurate engagement monitoring. As self-reported engagement – as synthesised by Donkin et al. (2011) – is an unreliable indicator of actual usage behaviour (Dwyer et al., 2024; Flett et al., 2019), this may account for the weaker associations observed in web-based trials. Second, the nature of the interventions themselves likely contributes to this divergence. Web programs are generally designed for prolonged, structured sessions involving substantial text and psychoeducation, making other metrics like overall site exposure a more relevant outcome predictor (Donkin et al., 2011). In contrast, apps are built for brief, flexible interactions that allow users to practice specific therapeutic skills in real time, making activity completion a more meaningful and proximal indicator of engagement and clinical benefit (Mohr et al., 2019).

Results from our meta-analysis provide preliminary support for the hypothesis that higher engagement is linked to greater clinical benefit. When averaging across engagement and outcome variables, we observed a small but significant pooled effect size of *r* = 0.16 (95 % CI = 0.09, 0.21) with no heterogeneity (I^2^ = 0 %). This effect remained significant in various sensitivity analyses that adjusted for small-study bias, reporting bias, outliers, and risk of bias. Furthermore, significant pooled effects were observed when modelling different metrics of engagement (activity completions, number of sessions, time on app), symptom outcomes (depression, general anxiety, social anxiety), and in sensitivity analyses examining specific app characteristics, including CBT-only apps, guided and unguided formats, apps with symptom-tracking technology, and both stand-alone and adjunctive app interventions. While these results suggest that a potential dose-response relationship may apply across various contexts in mental health app trials, they should be interpreted with considerable caution, as many of the sensitivity analyses were based on a limited number of studies.

Broader clinical and research implications emerge from the current findings. The significant meta-analytic association observed suggests that user engagement to mental health apps might be an important therapeutic change mechanism, supporting continued efforts to develop and trial novel engagement strategies (Linardon and Fuller-Tyszkiewicz, 2020; Liu et al., 2025). Although small in magnitude, an effect of this size (*r* = 0.16) may still hold clinical relevance at the population level given the scalability and reach of app-based interventions; however, it also highlights that engagement alone is unlikely to drive meaningful clinical change in isolation. Several methodological factors may also help explain the modest strength of the association. These include restricted variability and skewed distributions in engagement metrics, the use of metrics (e.g., logins, session counts) that may not fully capture therapeutic engagement, the post hoc nature of most engagement-outcome analyses, and variability in intervention duration and timing of measurement. Other therapeutic factors such as the quality rather than quantity of engagement, degree of content personalization, digital working alliance, sudden shifts in emotional or cognitive processes (e.g., increased self-awareness or insight), user motivation, external support systems, or human relationships facilitated by apps may also prove to be important change mechanisms (Domhardt et al., 2025; Goldberg et al., 2022; Henson et al., 2019; Nahum-Shani et al., 2022). Maximising the clinical benefits of mental health apps will require future research to clarify the potential mechanisms of action, allowing for the purposeful design or refinement of interventions that prioritise the most effective therapeutic elements.

From a research perspective, the variability and suboptimal reporting of engagement-outcome associations in existing trials highlight the urgent need for improved reporting standards in this field. We noted that these associations are often examined *post hoc*, using multiple engagement metrics that are readily available but lack theoretical justification. This exploratory approach likely contributes to the inconsistent and limited reporting of relevant data in publications, which in turn hampers the ability to synthesise findings through meta-analytic techniques. Given the lack of progress in advancing standard engagement metrics for app research (Bradway et al., 2020; Nwosu et al., 2022), we recommend that the field work toward a consensus on which engagement metrics are most conceptually and clinically meaningful in the context of app-based interventions. Standardising these metrics will facilitate more consistent analyses across trials and enable the pooling of data in future meta-analyses to produce more precise and reliable estimates.

As an initial step, we suggest prioritising the reporting of number of activity completions and app logins, as these are currently the most commonly reported metrics, align well with the intended use of mental health apps (brief, repeated interactions throughout daily life), and are consistent with other reviews (Elkes et al., 2024; Forbes et al., 2023). Days of use may also prove to be a useful metric, given evidence that this variable may be less skewed than other usage metrics and may capture sustained engagement (Goldberg et al., 2025). We suggest that at a minimum, all studies testing mental health apps report the correlation between the number of activities completed, app sessions, logins, and days of use with pre-post and pre-follow-up change in the study’s primary mental health outcome. For instances where engagement metrics are highly skewed (e.g., >2; Curran et al., 1996), it may be valuable to dichotomize the sample into high and low usage categories and to report between-group comparisons on change in the primary outcome. These between-group comparisons could be converted into correlation coefficients in future meta-analyses. Furthermore, reporting all relevant statistical tests in-text or in supplementary materials, or making engagement data available via open science repositories, will enhance transparency and reproducibility, ultimately strengthening the evidence base (Nosek et al., 2015). It will also be important that authors do not selectively report only those associations that are significant. Preregistering hypotheses related to associations between engagement and symptoms may help guard against potential publication bias.

If data are also available to researchers, it would be useful to report engagement metrics that are more proximal to the therapeutic process. Examples include time spent engaging with core skill-building modules, completion of evidence-based therapeutic exercises (e.g., cognitive restructuring, behavioural activation tasks), frequency of mood tracking completions, and active use of in-the-moment coping or emotion regulation tools. These indicators may more accurately capture the quality and therapeutic relevance of app use, providing a stronger signal of clinically meaningful engagement than broader metrics like logins or downloads. Including such process-oriented metrics alongside standard usage indicators would help clarify which types of engagement are most strongly linked to treatment response and could guide the development of more targeted engagement strategies in future interventions. Equally important, reporting these associations regardless of whether they reach statistical significance is critical for building a cumulative evidence base. Non-significant findings provide essential information that can refine theory, reduce publication bias, and strengthen future meta-analytic estimates of engagement-response relationships.

The current findings must be interpreted within the context of its limitations. First, the number of studies available for analysis was low, so the associations identified should be viewed as preliminary and with caution until more trials are conducted. Second, because dose-response associations are often explored *post hoc* and are not typically the primary focus of trials, there is still a possibility that some failed to report or mention non-significant results. In theory, all studies testing apps for depression and anxiety that gathered objective usage data could have reported the association between engagement and symptoms. Consequently, relevant studies may have been inadvertently excluded from this review, introducing a potential source of reporting bias. Due to the relatively modest number of available studies, we were also not able to apply newer publication bias assessment methods (e.g., PET-PEESE). A final limitation is that we only examined associations between engagement and symptoms. Thus, our results are ultimately only correlational in nature. It is possible that there is at least some degree of reverse causation where individuals who are benefitting more from a mental health app use the app more frequently, rather than the reverse. Future trials that randomly assign participants to varying engagement dosages will be essential to determine whether greater engagement causes improvement.

In conclusion, the current review is the first to synthesize empirical research investigating associations between engagement and clinical outcomes in randomized trials of mental health apps targeting depression and anxiety. We identified preliminary evidence of a small but significant dose-response relationship, which remained stable in various sensitivity analyses that modelled different engagement metrics, symptom outcomes, and app features. However, reporting was highly variable, highlighting the urgent need for the field to establish consensus on which theory-driven engagement metrics should be prioritised in future research. Standardising these metrics will not only enable more rigorous meta-analytic synthesis but also inform the design of mental health apps that optimise meaningful user engagement and therapeutic outcomes.

## Figures and Tables

**Table 1:** Characteristics of included studies.

Author	Sample	App features (n)	Control (n)	RoB	Outcome variable	Engagement metric(s)
Araya et al. (2021) – Trial 1**	Depression (PHQ-9 ≥ 10)	CONEMO (657) CBT Guided Symptom monitoring: NR Chat-bot: NR Format: Stand-alone	Usual care (655)	+ + - SR +	Depression (50 % decrease in PHQ-9)	Activities completed (3 levels) Zero (ns) 1–10 (ns) 11–27 (ns)
Araya et al. (2021) – Trial 2	Depression (PHQ-9 ≥ 10)	CONEMO (657) CBT Guided Symptom monitoring: NR Chat-bot: NR Format: Stand-alone	Usual care (655)	+ + - SR +	Depression (50 % decrease in PHQ-9)	Activities completed (3 levels) Zero (+) 1–10 (ns) 11–27 (ns)
Bastiaansen et al. (2022)	Depression (interview)	Do-Module (55) Non-CBT Guided Symptom mon: yes Chat-bot: NR Format: adjunct Think Module (55) Non-CBT Guided Symptom mon: yes Chat-bot: NR Format: adjunct	Usual care (51)	? ? – SR +	Depression (IDS-SR)	Activities completed (2 levels) (+) Compliant (≥75 % completion rate) Not compliant (< 75 % completion rate)
Bell et al. (2023) *	Depression & anxiety (PHQ-8 ≥ 10 and GAD-7 ≥ 10)	Mello (29) CBT Unguided Symptom mon: yes Chat-bot: NR Format: stand-alone	Waitlist (26)	+ + - SR +	Depression (PHQ-8) General anxiety (GAD-7)	Activities commenced (ns for dep & anx)* Check-ins completed (ns for dep & anx)* App use days (ns for dep & anx)* Proportion of app use days (ns for dep & anx)
Catuara-Solarz et al. (2022) **	Anxiety (GAD-7 score 5–18)	Foundations (95) CBT Unguided Symptom mon: NR Chat-bot: NR Format: stand-alone	Waitlist (95)	+ + – SR –	General anxiety (GAD-7)	App use days (ns) Time of app use (ns) Activities completed (ns)
Donker et al. (2019) reported in Donker et al. (2020)	Specific phobia (AQ ≥ 45.45)	Zerophobia (96) CBT Unguided Symptom mon: yes Chat-bot: NR Format: stand-alone	Waitlist (97)	+ + – SR +	Acrophobia symptoms (AQ)	Time of app use (+) Activities completed (ns)
Graham et al. (2020) **	Depression & anxiety (PHQ-8 ≥ 10 and GAD-7 ≥ 8)	IntelliCare (74) CBT Guided Symptom mon: yes Chat-bot: NR Format: stand-alone	Waitlist (72)	+ ? – + +	Depression (PHQ-8) General anxiety (GAD-7)	Number sessions completed * (ns for dep & anx) Time to last use (ns for dep & anx) App use days * (ns for dep & anx)
Greer et al. (2019)	Anxiety (HADS > 7)	Not names (72) CBT Unguided Symptom mon: NR Chat-bot: NR Format: stand-alone	Placebo (73)	+ ? + + +	General anxiety (HAM-A) Depression (HADS-D)	Number sessions completed (ns anx & + dep) Compliant (≥6 sessions) Not compliant (> 6 sessions)
Guo et al. (2020) reported in Li et al. (2022)	Depression (CES-*D* ≥ 16)	Run4Love (150) CBT Unguided Symptom: NR Chat-bot: NR Format: stand-alone	Information resources (40)	+ ? – SR +	Depression (CES-D)	Number activities completed (2 levels) (+) Compliant (average 74 % completed activities) Not compliant (average of 15 % completed activities)
Kulikov et al. (2023) *	Depression (self-identified)	Spark Direct (35) CBT Unguided Symptom mon: yes Chat-bot: yes Format: adjunct	Placebo (25)	+ ? + SR –	Depression (PHQ-8)	Number activities completed (+)*
Lacey et al. (2023) *	Specific phobia (BSSSP ≥ 4)	oVRcome (63) CBT Unguided Symptom mon: NR Chat-bot: NR Format: stand-alone	Waitlist (63)	+ ? – SR –	Phobic symptoms (SMSP)	Hours of app use (ns)*
Mantani et al. (2017) reported in Furukawa et al. (2018)*	Depression (interview)	Kokora (81) CBT Guided Symptom mon: yes Chat-bot: NR Format: adjunct	Antidepressants (83)	+ + + SR +	Depression (≥ 4 points change on PHQ-9)	Number sessions completed (ns)* Days to complete one session (ns) Time spent (ns)* Number mind map activities (ns)* Number behavioral activation activities (+)* Number cognitive restructuring activities (+)*
Miklowitz et al. (2023) **	Mood disorder (interview)	My Coach Connect Not CBT Guided Symptom mon: yes Chat-bot: NR Format: adjunct	Placebo (32)	+ ? + + ?	Depression/mania (PSRS)	Number of days with completed activities (ns) Number of weeks with completed activities (ns)
Moberg et al. (2019) *	Depression or anxiety (PHQ-9 or GAD-7 score 5–14)	Pacifica (253) CBT Unguided Symptom mon: yes Chat-bot: NR Format: stand-alone	Waitlist (247)	? ? – SR +	Depression (PHQ-9) General anxiety (GAD-7)	Number of logins (ns dep & anx)* Number thought records complete (ns dep & anx)* Number goals complete (ns dep & anx) Number relaxations complete (ns dep & anx) Number community activities complete (ns dep & anx)
Mohr et al. (2019) *	Depression or anxiety (PHQ-9 ≥ 10 or GAD-7 ≥ 8)	IntelliCare (74, 76, 75, 76) CBT Guided/guided/unguided/ unguided Symptom mon: NR Chat-bot: NR Format: stand-alone	-	? ? + SR +	Depression (PHQ-9) General anxiety (GAD-7)	Number app sessions (+ dep, ns anx) * Time to last app use (+ dep, ns anx) Number app downloads (+ dep & anx) *
Newman et al. (2021) **	Anxiety (GAD-Q criteria)	Not named (50) CBT Guided Symptom mon: yes Chat-bot: NR Format: stand-alone	Waitlist (50)	+ + - SR +	General anxiety (DASS-A, PSWQ & STAI-T composite)	Number app sessions (ns) Number messengers sent (ns) Number messengers received (ns) Number app visits (ns) Time spent on app (ns)
Oh et al. (2020) **	Panic disorder (interview)	Todaki (23) CBT Unguided Symptom: yes Chat-bot: yes Format: stand-alone	Information resources (22)	? ? – SR –	Panic symptoms (PDSS)	Time spent on app (ns) Days of app use (ns)
Raevuori et al. (2021) *	Depression (interview)	Meru Health Program (63) CBT Guided Symptom mon: NR Chat-bot: NR Format: Adjunct	Usual care (61)	+ + - + +	Depression (PHQ-8) Generalized anxiety (GAD-7)	Minutes of mindfulness practice (ns for dep & anx)* Number of messages sent to therapist (ns for dep & anx)
Roy et al. (2021)	Anxiety (interview)	Unwinding Anxiety (32) Non-CBT Guided Symptom mon: yes Chat-bot: NR Format: Adjunct	Usual care (33)	+ + - SR –	Generalized anxiety (GAD-7)	Number modules completed (+)
Saulnier et al. (2023) *	Social anxiety (ASI ≥ 6)	Boast (19) CBT Guided Symptom mon: yes Chat-bot: NR Format: stand-alone	Waitlist (17)	? ? – SR –	Social anxiety (ASI-3)	Number activities completed (+)* Number of completed EMA prompts (ns)
Schwob and Newman (2023)	Social anxiety (interview)	ImExposure (43) CBT Unguided Symptom mon: yes Chat-bot: NR Format: stand-alone	Placebo (39)	+ ? + SR +	Social anxiety (Composite SIAS & SPDQ)	Number activities completed (2 levels) (+) * Compliant (≥ 3 times daily) Not compliant (< 3 times daily)
Six et al. (2022) *	Depression (PHQ-8 ≥ 5)	AirHeart (45, 49) CBT Unguided/unguided Symptom mon: yes/yes Chat-bot: NR Format: stand-alone	-	+ ? + SR ?	Depression (PHQ-9)	Number activities (journals) completed (ns)*
Stiles-Shields et al. (2018) **	Depression (PHQ-9 ≥ 10 & QIDS ≥ 11)	Boost Me (10) CBT Guided Symptom mon: yes Chat-bot: NR Format: stand-alone Thought Challenger (1) CBT Guided Symptom mon: NR Chat-bot: NR Format: stand-alone	Waitlist (10)	+ + - SR –	Depression (PHQ-9)	Number of app logins (ns) Number activities completed (ns)
Colleen Stiles-Shields et al. (2024)**	Depression or anxiety (PHQ-9 ≥ 10 or GAD-7 ≥ 8)	Vira (65) Not CBT Guided Symptom mon: yes Chat-bot: NR Format: Stand-alone	Placebo (65)	? ? + - +	Depression (PHQ-9) General anxiety (GAD-7)	Number of app logins (ns for dep & anx) Number of coaching interactions (ns for dep & anx)
Stolz et al. (2018) *	Social anxiety (interview)	Not named (60) CBT Guided Symptom mon: yes Chat-bot: NR Format: stand-alone	Waitlist (30) Web treatment (60)	+ ? - + +	Social anxiety (SPS, SIAS & LSAS composite)	Number of lessons completed (+) Number of hours spent in app (ns) * Number of separate sessions (ns)* Number of app clicks (ns) Number of relaxation exercises (ns) * Number of fear-provoking exercises (ns) * Number recorded helpful thoughts (ns) * Number exposure exercises (+) * Number mails written by patients (ns) Number of characters written by patients in mails (ns)
Tighe et al. (2017) reported in Tighe et al. (2020)**	Depression (PHQ-9 ≥ 10 or K-10 ≥ 25)	iBobbly (31) Not CBT Guided Symptom mon: yes Chat-bot: NR Format: stand-alone	Waitlist (10)	+ ? – SR +	Depression (PHQ-9)	Time spent on app (ns)
Yatziv et al. (2024)	Depression (interview)	MoodVille (87) Not CBT Unguided Symptom: NR Chat-bot: NR Format: stand-alone	Waitlist (30)	? ? – SR –	Depression (MADRS)	Days of app use (2 levels) (+) Compliant (≥ 4 days per week) Not compliant (< 4 days per week)
Zuccolo et al. (2024)	Depression (interview)	Motherly (132) CBT Unguided Symptom: Yes Chat-bot: NR Format: stand-alone	Placebo (132)	+ + + SR +	Depression (EPDS)	Number activities completed (ns)

* indicates that the study/engagement metric was included in the meta-analysis. NR = not reported; PHQ-9 = Patient Health Questionnaire; EPDS = Edinburgh Postnatal Depression Scale; MADRS =Montgomery–Åsberg Depression Rating Scale; GAD-7 = Generalized Anxiety Disorder Scale; SPS = Social Phobia Scale; SIAS = Social Interaction Anxiety Scale; LSAS = Liebowitz Social Anxiety Scale; SPDQ = Social Phobia Diagnostic Questionnaire; PDSS= Panic Disorder Severity Scale; STAI-Strait-Trait Anxiety Scale; DASS = Depression Anxiety Stress Scale; AQ = Acrophobia Questionnaire; HADS = Hospital Anxiety Depression Scale; CES-D = Center for Epidemiologic Studies Depression Scale; IDS-SR = Inventory of Depressive Symptomatology – Self-Report.

Ns = non-significant; + = greater engagement associated with greater symptom change.

For RoB, ? = unclear, - = high risk, + = low risk. In order, sequence generation, allocation concealment, blinding of participants; use of self-report; and appropriate handling of missing data.

** indicates studies for which did not provide data to calculate effect sizes, but were coded as *r* = 0 in the sensitivity analyses.

**Table 2:** Summary of Engagement Metric–Symptom Outcome Associations Across Studies.

Engagement Metric	Depression Sig/non-sig	General anxiety Sig/non-sig	Social anxiety Sig/non-sig	Specific phobia Sig/non-sig	Panic Sig/non-sig	Total Sig/non-sig
In-app activity/tasks completions	5/18	0/10	3/5	0/1	-	8/34
Days of app use	1/3	0/3	-	-	0/1	1/7
Time of app use	0/2	0/2	0/1	1/1	0/1	1/7
Number of sessions/app opens	2/5	1/7	0/1	-	-	3/13
Other	1/5	1/5	1/2	-	-	3/12

Significant relationships were all in the expected direction (higher engagement linked with greater improvement).

In-app activities comprised the following operationalisations: number check-ins, number of mind maps, behavioral activation, and cognitive restructuring activities, journal frequency, number of thought records, number of goals completed, number of relaxation exercises, number of community activities, number of messages sent to coach, total minutes of mindfulness practice, number of EMA prompts completed.

Time of app use comprised the following operationalisations: time per day average; total time of use; total hours of use; time spent on a session.

Number of sessions/app opens comprised the following operationalisations: number of app sessions, number of logins, number of downloads, number of visits, number of opens.

“other” comprised: proportion of days using the app; time to last use, days to complete a session, number of weeks with logged activity practice, number of messaged received from coach; number modules completed; coaching interaction frequency; number of clicks; number of characters written by patients.

**Table 3:** Results from the meta-analysis.

Analysis	*k*	*r* (95 % CI)	*p*	I^2^
Total pooled effect	13	.16 (0.09, 0.21)	<0.001	0 %
Assumed *r* = 0 for non-sig. unreported studies	21	.11 (0.06, 0.16)	<0.001	0 %
Trim-and-Fill estimate	2 trimmed	.14 (0.07, 0.21)		
Outliers removed	13	.16 (0.09, 0.21)	<0.001	0 %
High risk of bias trials removed	12	.15 (0.09, 0.21)	<0.001	0 %
Engagement metrics				
Number of days of use	2	.11 (−0.15, 0.37)	.395	37 %
Number of completed tasks/activities	9	.19 (0.11, 0.27)	<0.001	0 %
Number of app sessions	6	.12 (0.05, 0.20)	.001	0 %
Time spent on the app	3	.17 (0.01, 0.31)	.027	0 %
Symptom outcomes				
Depression	9	.14 (0.07, 0.21)	<0.001	0 %
Generalized anxiety	6	.09 (0.01, 0.18)	.017	0 %
Social anxiety	4	.25 (0.08, 0.40)	.003	31 %
App features				
CBT app	12	.16 (0.09, 0.22)	<0.001	5 %
Guided app	5	.18 (0.02, 0.33)	.026	33 %
Unguided app	8	.15 (0.08, 0.22)	<0.001	0 %
Symptom monitoring features	8	.17 (0.07, 0.26)	.001	22 %
Stand-alone	10	.15 (0.08, 0.22)	<0.001	77 %
Augmented treatment	3	.19 (0.04, 0.34)	.012	0 %

## Footnotes

1 Mixed anxiety and depression refer to samples comprising individuals with elevated symptoms of depression, anxiety, or both, even if these conditions did not necessarily co-occur within the same individual.
